# Death receptor 5 (*DR5*) and a 5-gene apoptotic biomarker panel with significant differential diagnostic potential in colorectal cancer

**DOI:** 10.1038/srep36532

**Published:** 2016-11-09

**Authors:** Marina Devetzi, Vivian Kosmidou, Margarita Vlassi, Iraklis Perysinakis, Chrysanthi Aggeli, Theodosia Choreftaki, Georgios N. Zografos, Alexander Pintzas

**Affiliations:** 1 Laboratory of Signal Mediated Gene Expression, Institute of Biology, Medicinal Chemistry and Biotechnology, National Hellenic Research Foundation, Athens, Greece; 2 3rd Department of Surgery, General Hospital of Athens “G. Gennimatas”, Athens, Greece; 3 Department of Pathology, General Hospital of Athens “G. Gennimatas”, Athens, Greece

## Abstract

High expression of Inhibitor of apoptosis proteins (IAPs) has been related to colorectal cancer (CRC) progression, resistance to treatment and poor prognosis. TRAIL (TNF-related apoptosis-inducing ligand) through its receptors DR4 (TRAIL-R1) and DR5 (TRAIL-R2) can selectively induce cancer cell apoptosis. The mRNA expression of *DR4*, *DR5*, *c-IAP1*, *c-IAP2*, *XIAP* and *BIRC5*/Survivin genes was examined in 100 paired (cancerous-normal) colorectal tissue specimens by real-time PCR, 50 of which were *KRAS* wild-type and 50 *KRAS*-mutant. *DR5*, *XIAP* and *BIRC5*/Survivin genes are significantly up-regulated (*p* < 0.0001, *p* = 0.012 and *p* = 0.0003, respectively), whereas *c-IAP1* and *c-IAP2* genes are significantly down-regulated at mRNA and protein levels in CRC (*p* < 0.0001 for both). ROC analyses showed that *DR5*, *cIAP1* and *cIAP2* expression has discriminatory value between CRC and normal tissue (AUC = 0.700, *p* < 0.0001 for *DR5*; AUC = 0.628, *p* = 0.011 for *cIAP1*; AUC = 0.673, *p* < 0.0001 for *cIAP2*). Combinatorial ROC analysis revealed the marginally fair discriminatory value of 5 genes as a panel (AUC = 0.685, *p* < 0.0001). Kaplan-Meier survival curves revealed significant association of *cIAP2* down-regulation in CRC with lower overall survival probability of CRC patients (*p* = 0.0098). *DR5*, *BIRC5*/Survivin, *XIAP*, *c-IAP1* and *c-IAP2* mRNA expression are significantly deregulated in CRC and could provide a panel of markers with significant discriminatory value between CRC and normal colorectal tissue.

Colorectal cancer (CRC) is one of the most frequently diagnosed types of cancer in western industrialized countries^1^. It is the second most common malignancy diagnosed in women, after breast cancer, and the third most common in men after prostate and lung cancer. CRC is a preventable disease in most cases due to the introduction of CRC screening, primarily in the form of colonoscopy^2^. For the time being, surgical resection of the tumor, followed by adjuvant chemotherapy, remains the prominent choice for treatment. Despite surgery, 45% of patients ultimately die of distant metastases; 5-year overall survival decreases from approximately 90% for stage I patients to about 8% for stage IV. The stage of the disease at the time of diagnosis is crucial to survival; unfortunately, in a large number of cases CRC is diagnosed in advanced stages^3^. However, approximately 20–25% of CRC patients present with liver metastases at the time of initial diagnosis and another 20–25% will develop metastases during disease progression^4^. It is obvious that early detection is critical, however the available methods for screening encounter several difficulties in meeting that expectation. It is therefore of great importance that new and improved methods are applied. During the last decades, there has been an extensive effort for the discovery of novel tissue- and serum-based diagnostic, prognostic and predictive biomarkers for CRC, due to the fact that current markers in use lack specificity and sensitivity^5^,^6^.

One of the most frequently mutated genes in CRC is the *KRAS* oncogene, component of the RAS/RAF/MEK/ERK signaling pathway downstream of EGFR (Epidermal Growth Factor Receptor), which is a significant regulator of cell growth, proliferation, differentiation and apoptosis. *KRAS* is found mutated in approximately 40% of CRC cases, mainly in the codons 12 (wild-type GGT) and 13 (wild-type GGC) of exon 1, with demonstrated capacity as major predictive markers of resistance to treatment with anti-EGFR monoclonal antibodies in metastatic CRC. Therefore, the effectiveness of treatment is dependent on the mutation status of *KRAS* in CRC^7^,^8^,^9^.

Apoptotic cell death is important for the homeostasis of normal colorectal epithelial cells, which are generated from stem cells at the base of the crypt region via their differentiation and migration to the epithelial surface, where they eventually die by apoptosis and are discarded in the lumen of the colon^10^,^11^. Imbalance in cell death signaling is implicated in CRC, since it is well known that the failure of apoptotic cell death pathways constitutes a crucial process for the survival of cancer cells and a leading cause of resistance to current therapeutic approaches^12^,^13^.

TRAIL (TNF-related apoptosis-inducing ligand) is a cytokine best known for its ability to selectively induce apoptosis in cancer cells while sparing most normal cells, which made it a rather attractive potential chemotherapeutic agent. TRAIL has been shown to induce apoptosis in malignant cells both *in vitro* and in pre-clinical models of cancer^14^,^15^,^16^,^17^. However, TRAIL therapy has a major limitation due to the fact that a large number of cancers become resistant to TRAIL and escape from immunosurveillance mechanisms^15^,^18^,^19^. The proapoptotic TRAIL receptors DR4 (TRAIL-R1) and DR5 (TRAIL-R2) not only trigger apoptosis in TRAIL-sensitive cells, but also activate survival pathways in tumor cells that resist the induction of cell death upon exposure to TRAIL^19^. Moreover, it has been reported that there is a marked increase in sensitivity of TRAIL-induced apoptosis during transition from colorectal adenoma to CRC^20^,^21^. It was recently shown that constitutive signaling from DR5 (TRAIL-R2) promotes migration and invasion in a cancer cell-autonomous manner^22^. As a biomarker, DR5 was found significantly up-regulated in stage II and III CRC by immunohistochemistry but without prognostic significance^23^. Bavi *et al.* reported that DR4 was also significantly up-regulated in CRC and adenomas by immunohistochemistry and was associated with a less aggressive phenotype characterized by early stage disease, whereas DR5 was associated with a microsatellite stable (MS–S/L) phenotype and with absence of KRAS mutations in a Middle-Eastern population^24^.

Inhibitor of apoptosis proteins (IAPs) are eight anti-apoptotic human proteins characterized by the presence of one to three baculoviral IAP repeats (BIR) domains. The most extensively studied IAP protein family members are cIAP1, cIAP2, XIAP, ML-IAP and BIRC5/Survivin^11^,^25^. Functionally, IAPs have been shown to interact with multiple regulators of both the extrinsic (death receptor) and the intrinsic (mitochondrial) apoptotic pathways and inhibit the activation of caspases (caspases 3, 7, 8, 9) directly (XIAP) or indirectly (cIAP1, cIAP2) via binding to SMAC/DIABLO (Second mitochondria-derived activator of caspase, SMAC) endogenous inhibitor of XIAP. In addition, cIAP1 and cIAP2 are crucial activators of the canonical NF-κB (Nuclear factor-kappa B, NF- κB) signaling pathway by TNF (Tumor necrosis factor, TNF) and negative regulators of the non-canonical NF-κB signaling^26^,^27^. Therefore, IAPs act as modulators of cell viability by inducing pro-survival signaling pathways.

In cancer, IAPs are found to be overexpressed in several malignancies, including CRC, and their action has been reported to enhance cancer cell resistance to apoptotic death stimuli. Increased IAP expression has been related to tumorigenesis, progression of cancer, resistance to treatment and poor prognosis^12^,^28^,^29^. Hence, IAP proteins could represent promising biomarkers in CRC and/or targets for therapeutic intervention.

The aim of the present study is to investigate the mRNA expression profile of the death receptor genes *DR4* (TRAIL-R1) and *DR5* (TRAIL-R2), as well as *IAP* genes *cIAP1*, *cIAP2*, *XIAP* and *BIRC5*/Survivin in CRC and normal tissue samples and to evaluate their potential diagnostic and prognostic significance, as well as their putative association with *KRAS* mutation status in CRC. *DR5, BIRC5*/Survivin*, XIAP, cIAP1* and *cIAP2* gene expression is found significantly deregulated in CRC at the mRNA level and could provide a 5-gene panel of markers with significant discriminatory value between CRC and normal colorectal tissue.

## Results

In the current study we examined 100 cancerous and 100 normal paired colorectal tissues for the mRNA expression levels of *DR5, DR4* and *BIRC5*/Survivin, *XIAP, cIAP1*, *cIAP2* (IAPs) genes, using quantitative real-time PCR analysis. Data were normalized to *GAPDH* and *HPRT* mRNA expression levels. All samples yielded analyzable data. Out of the 100 CRC tissues included in the study, 50 were *KRAS* wild-type and 50 were *KRAS*-mutant tissues, harboring the activating *KRAS-G13D*, *KRAS-G12D* and *KRAS-G12V* mutations. The descriptive statistics regarding the expression status of all genes under study in patients with primary CRC are presented in Table 1. Follow-up information (ranging 1–99 months) was available for 65 patients and included survival status (alive or deceased) along with the dates of the events and cause of death. During respective follow-up period, 23 patients (35.4%) had passed away.

### *DR5* is significantly up-regulated in CRC

**Figure 1:**
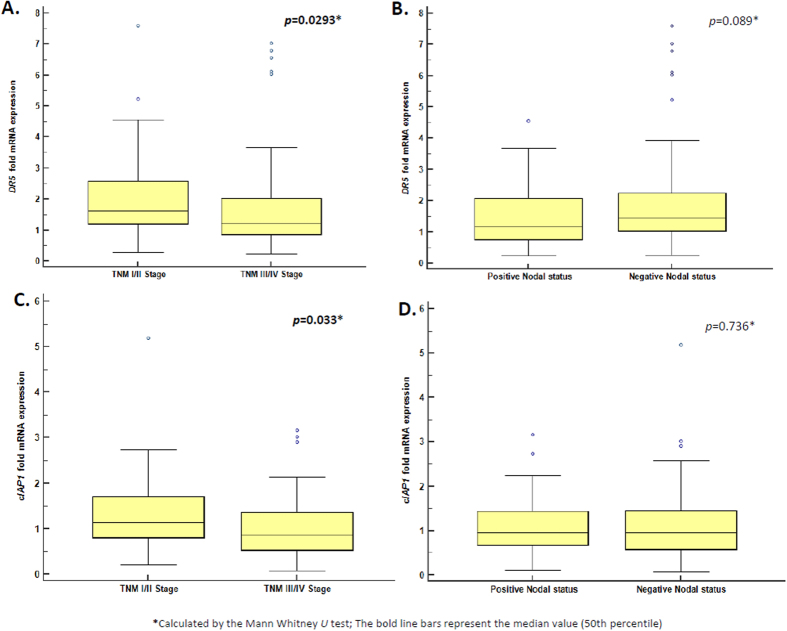
(**A**) Box plots show the significantly increased mRNA expression of *DR5* in TNM stage classified CRC tissues. Quartiles (25th, 50th-median, 75th percentiles) are within the box. The horizontal lines indicate the medians. The upper and nether horizontal lines indicate the 90th and 10th percentiles, respectively, (**B**) Box plots show the significantly increased mRNA expression of *DR5* in lymph node invasion status classified CRC tissues, (**C**) Box plots show the significantly decreased mRNA expression of *cIAP1* in TNM stage-classified CRC tissues. (**D**) Box plots show the significantly decreased mRNA expression of *cIAP1* in lymph node invasion status-classified CRC tissues.

**Figure 2:**
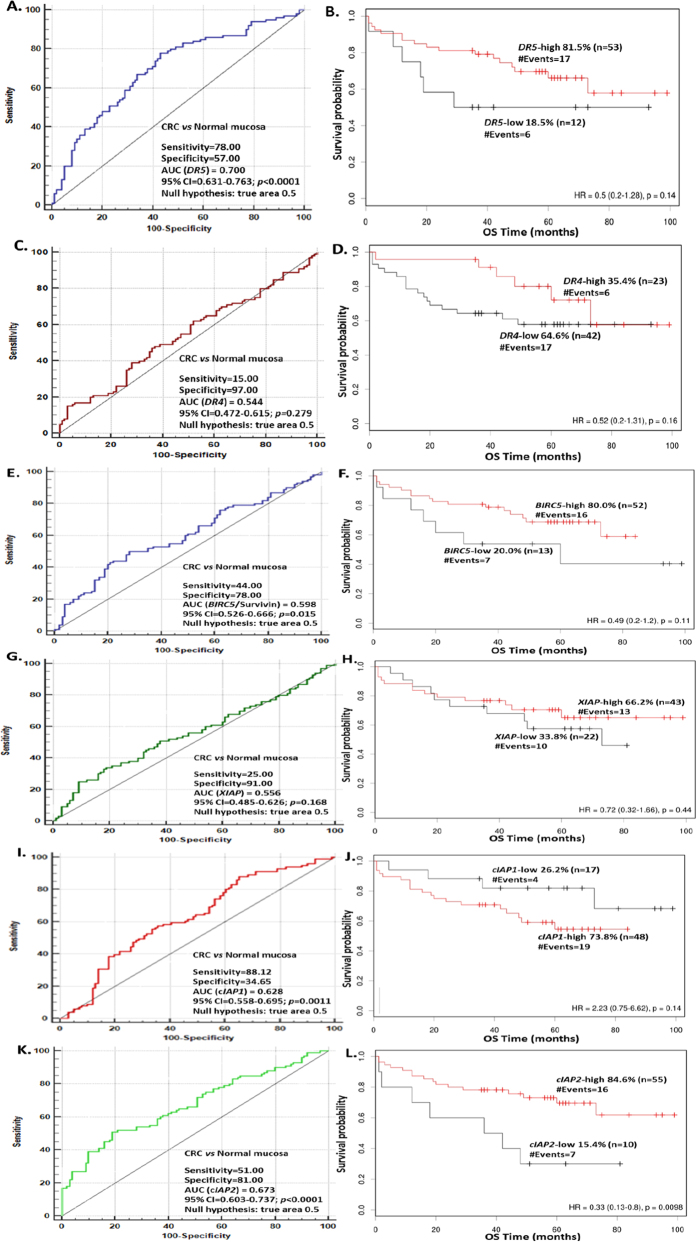
ROC curve analyses indicating the discriminatory potential of *DR5* (**A**), *DR4* (**C**), *BIRC5*/Survivin (**E**), *XIAP* (**G**), *cIAP1* (**I**) and *cIAP2* (**K**) mRNA expression levels in CRC and normal tissues. Kaplan-Meier curves for OS of CRC patients with *DR5-*high and *DR5-*low (**B**), *DR4-*high and *DR4-*low (**D**), *BIRC5*-high and *BIRC5*-low (**F**), *XIAP*-high and *XIAP*-low (**H**), *cIAP1*-high and *cIAP1*-low (**J**) and *cIAP2*-high and *cIAP2*-low (**L**) gene expression.

Death receptor gene *DR5* (TRAIL-R2) was found to be over-expressed in CRC tissues compared to normal pairs. *DR5* showed very significant higher mRNA expression levels in CRC tissues (mean ± SEM: 1.915 ± 0.16 fold expression units) compared to normal counterparts (mean ± SEM: 1.174 ± 0.11 fold expression units) (*p* < 0.0001), as calculated by the Wilcoxon Signed Ranks test (Tables 1 and 2). As presented in Table 1, *DR5* mRNA expression was found to be 1.58-fold higher in CRC specimens than in normal ones, according to median –fold expression value. We also observed that the increase of *DR5* mRNA expression was significantly higher in low TNM stage (I/II) colorectal tumors (*p* = 0.0293) compared to high TNM stage (III/IV) ones (Fig. 1A) with the use of Mann Whitney *U* test. The increase of *DR5* mRNA expression in CRC was not significantly different between groups of patients classified according to lymph node invasion status (*p* = 0.089) (Fig. 1B). In order to evaluate the discriminatory value of *DR5* mRNA expression profile between cancer and normal tissue, we performed ROC analysis. ROC curves were constructed for *DR5* expression levels by plotting sensitivity versus (1-specificity). The ROC curve (Fig. 2A) illustrates fair discriminatory power of *DR5* mRNA expression levels in distinguishing cancer from normal tissues, with strong statistical significance (AUC = 0.700; 95%CI = 0.631–0.763; *p* < 0.0001). Survival curves determined by the Kaplan-Meier method for overall survival (OS) demonstrated that *DR5* expression is not significantly associated with OS of CRC patients (*p* = 0.140) (Fig. 2B).

*DR4* (TRAIL-R1) mRNA expression was also elevated in CRC (mean ± SEM: 1.926 ± 0.13 fold expression units) compared to normal tissues (mean ± SEM: 1.661 ± 0.08 fold expression units) (Table 1), but not in a statistically significant manner (*p* = 0.548), as calculated by the Wilcoxon Signed Ranks test (Table 2). *DR4* mRNA expression was found 1.15-fold higher in CRC specimens than in normal ones, according to median –fold expression value (Table 1). The increase of *DR4* mRNA expression in CRC was not significantly different between groups of patients classified according to TNM stage or lymph node invasion status (Suppl. Figure 1A,B, respectively). As expected, ROC curve analysis for *DR4* mRNA expression levels (Fig. 2C) did not show discriminatory power in distinguishing cancer from normal tissues (AUC = 0.544; 95%CI = 0.472–0.615; *p* = 0.279). Kaplan-Meier survival curves for OS demonstrated that *DR4* expression is not significantly associated with OS of CRC patients (*p* = 0.160) (Fig. 2D).

### *BIRC5*/Survivin and *XIAP* are significantly up-regulated in CRC

*BIRC5*/Survivin gene, of the IAP gene family, was significantly up-regulated in the 100 CRC tissues examined (mean ± SEM: 0.631 ± 0.06 fold expression units) compared to normal counterparts (mean ± SEM: 0.436 ± 0.04 fold expression units) at the mRNA level (*p* = 0.0003), as calculated by the Wilcoxon Signed Ranks test (Tables 1 and 2). Differential mRNA expression of *BIRC5*/Survivin was found 1.35-fold higher in CRC specimens than in normal ones, according to median –fold expression value (Table 1). The increase of *BIRC5*/Survivin mRNA expression in CRC was not significantly different between groups of patients classified according to TNM stage or lymph node invasion status (Suppl. Figure 1C,D, respectively). ROC curve analysis for *BIRC5*/Survivin expression levels (Fig. 2E) demonstrates marginal discriminatory power in distinguishing cancer from normal tissues with statistical significance (AUC = 0.598; 95%CI = 0.526–0.666; *p* = 0.015). Kaplan-Meier survival curves for OS demonstrated that *BIRC5*/Survivin expression is not significantly associated with OS of CRC patients (*p* = 0.110) (Fig. 2F).

*XIAP* gene, also of the IAP gene family, is also significantly up-regulated in the 100 CRC tissues examined (mean ± SEM: 1.299 ± 0.10 fold expression units) compared to normal counterparts (mean ± SEM: 1.090 ± 0.09 fold expression units) at the mRNA level (*p* = 0.012), as calculated by the Wilcoxon Signed Ranks test (Tables 1 and 2). Differential mRNA expression of *XIAP* was found 1.18-fold higher in CRC specimens than in normal ones, according to median –fold expression value (Table 1). The increase of *XIAP* mRNA expression in CRC was not significantly different between groups of patients classified according to TNM stage or lymph node invasion status (Suppl. Figure 1E,F, respectively). ROC curve analysis for *XIAP* mRNA expression levels (Fig. 2G) did not reveal discriminatory power in distinguishing cancer from normal tissues (AUC = 0.556; 95%CI = 0.485–0.626; *p* = 0.168). Kaplan-Meier survival curves for OS demonstrated that *XIAP* expression is not significantly associated with OS of CRC patients (*p* = 0.440) (Fig. 2H).

### *cIAP1* and *cIAP2* are significantly down-regulated in CRC

*cIAP1* gene was found significantly down-regulated in CRC (mean ± SEM: 1.0922 ± 0.08 fold expression units) compared to normal tissue (mean ± SEM: 1.509 ± 0.09 fold expression units) at the mRNA level (*p* < 0.0001), as calculated by the Wilcoxon Signed Ranks test (Tables 1 and 2). Differential mRNA expression of *cIAP1* was 0.19-fold lower in CRC specimens than in normal ones, according to median –fold expression value (Table 1). The decrease of *cIAP1* mRNA expression in CRC was significantly observed in high TNM stage (III/IV) colorectal tumors compared to low TNM stage (I/II) ones (*p* = 0.033) (Fig. 1C), whereas it was not significantly different between groups of patients classified according to lymph node invasion status (*p* = 0.736) (Fig. 1D). ROC curve analysis for *cIAP1* mRNA expression levels (Fig. 2I) revealed poor discriminatory power in distinguishing cancer from normal tissues with strong statistical significance (AUC = 0.628; 95%CI = 0.558–0.695; *p* = 0.0011). Kaplan-Meier survival curves for OS demonstrated that *cIAP1* down-regulation in CRC is not significantly associated with OS patients (*p* = 0.140) (Fig. 2J).

Similarly, *cIAP2* gene was found significantly down-regulated in CRC (mean ± SEM: 5.652 ± 0.53 fold expression units) compared to normal tissue (mean ± SEM: 9.208 ± 0.93 fold expression units) at the mRNA level (*p* < 0.0001), as calculated by the Wilcoxon Signed Ranks test (Tables 1 and 2). Differential mRNA expression of *cIAP2* was 0.43-fold lower in CRC specimens than in normal ones, according to median –fold expression value (Table 1). The decrease of *cIAP2* mRNA expression in CRC was not significantly different between groups of patients classified according to TNM stage or lymph node invasion status (Suppl. Figure 1G,H, respectively). ROC curve analysis for *cIAP2* mRNA expression levels (Fig. 2K) revealed poor discriminatory power between cancer and normal tissues, although with very strong statistical significance (AUC = 0.673; 95%CI = 0.603–0.737; *p* < 0.0001). Kaplan-Meier survival curves for OS demonstrated that *cIAP2* down-regulation in CRC is significantly associated with lower survival probability and shorter OS of CRC patients (*p* = 0.0098) (Fig. 2L).

### Significant discriminatory value of a five-gene panel between CRC and normal colorectal tissue

**Figure 3:**
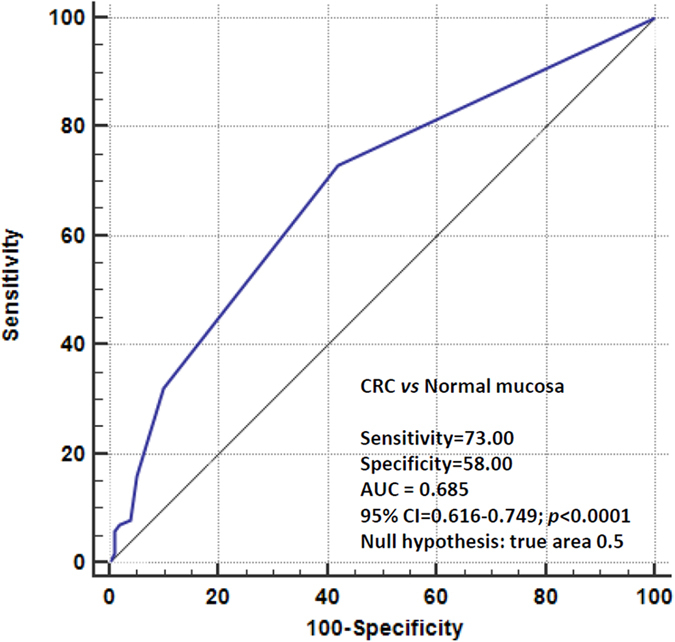
A 5-gene ROC curve analysis for the discrimination between CRC and normal tissues. Quantitative 5-gene PCR data sets were combined using logistic regression in a unique 5-gene assay and the resulting predicted probabilities were subjected to ROC curve analysis. Sensitivity, specificity, *p*-value and AUC values are enclosed.

Quantitative PCR demonstrated differential mRNA expression of 5 genes *DR5, XIAP, BIRC5*/Survivin*, cIAP1* and *cIAP2* (except *DR4*) between CRC and normal tissue. Data were combined using logistic regression and 5-gene panel ROC analysis was performed on all samples in order to evaluate overall discriminatory value of the 5 genes expression levels between CRC and normal tissues. As shown in Fig. 3, there is very significant marginally fair discriminatory value of the 5-gene panel between CRC and normal tissues (AUC = 0.685, 95%CI = 0.616–0.749, *p* < 0.0001). Thus, a 5-gene assay of *DR5* and *IAPs* could have differential diagnostic perspectives.

### Differential mRNA expression profile according to *KRAS* mutation status of CRC tissues

Since *KRAS* mutation status in CRCs is correlated with resistance to treatment with anti-EGFR monoclonal antibodies, the effort for novel therapeutic approaches, such as TRAIL-related therapeutics for *KRAS-* mutated CRCs is crucial. In the present study we investigated the association of mRNA expression levels of TRAIL receptors (*DR4* and *DR5*) and selected *IAP* genes with the presence of *KRAS* mutations in colorectal tumors. Our study group consisted of 50 *KRAS* mutated (*KRAS-G13D*, *KRAS-G12D*, *KRAS-G12V*) CRCs and 50 *KRAS* wild-type ones.

### *DR5* and *cIAP1* deregulation in CRC is significant independently of *KRAS* mutation status

**Figure 4:**
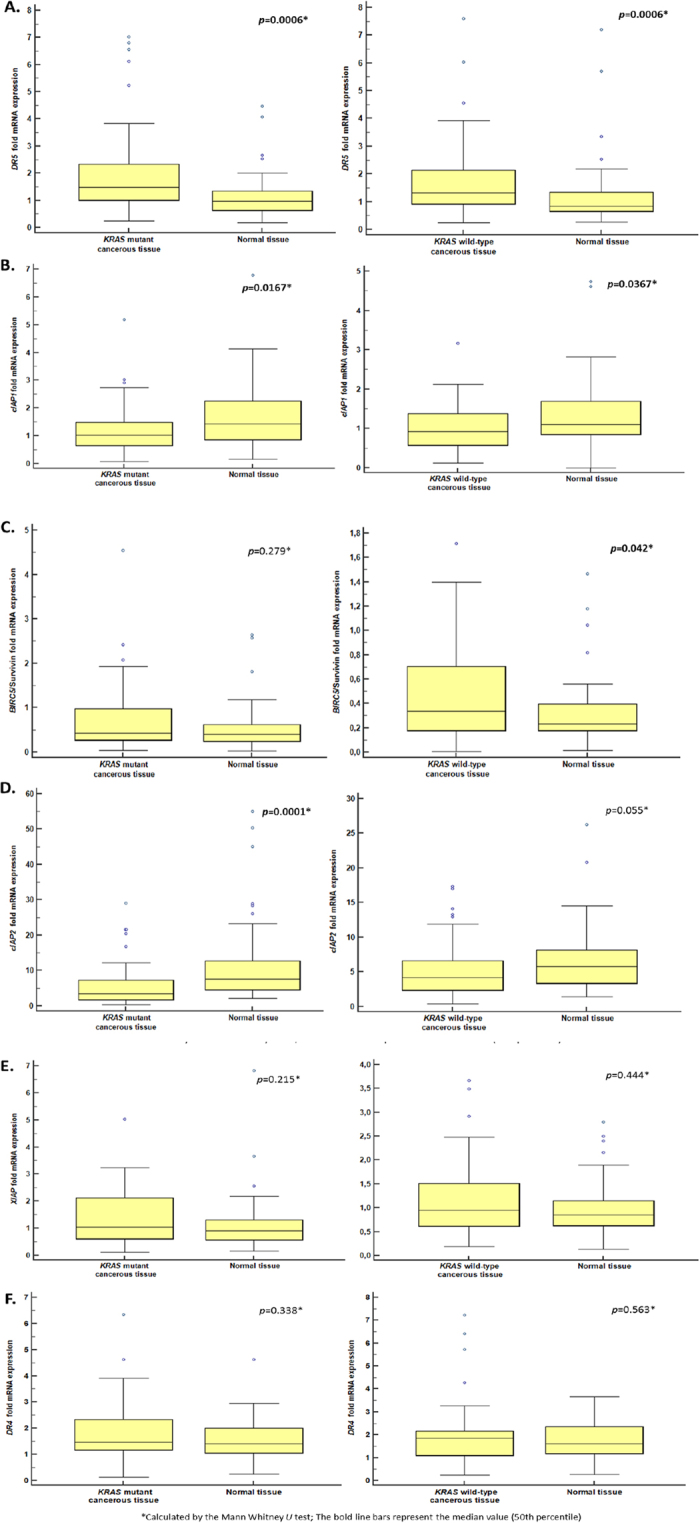
*DR5* and *IAPs* gene expression in *KRAS*-mutant (*KRAS-G13D*, *KRAS-G12D*, *KRAS-G12V*) and *KRAS* wild-type CRC tissues. (**A**) Box plots show the significant over-expression of *DR5* in CRCs regardless of *KRAS* mutation status, (**B**) Box plots show significant down-regulation of *cIAP1* in CRC tissues regardless of *KRAS* mutation status, (**C**) Box plots show the significant over-expression of *BIRC5*/Survivin in *KRAS* wild-type CRC tissues, but not in *KRAS*-mutant ones, (**D**) Box plots show significant down-regulation of *cIAP2* in *KRAS*-mutant CRC tissues, but not in *KRAS* wild-type ones, (**E**,**F**) Box plots show that the deregulation of *XIAP* and *DR4* mRNA expression in CRC is not significant in *KRAS* mutation status sybgroups of patients.

*DR5* mRNA expression was found significantly elevated in both *KRAS* mutant and *KRAS* wild-type CRC tissues with greater significance in *KRAS* mutant (*KRAS-G13D*, *KRAS-G12D*, *KRAS-G12V*) ones (*p* = 0.0006 for both) compared to normal pairs (Fig. 4A).

*cIAP1* mRNA expression did not show differentiation depending on *KRAS* mutation status of CRC tissues, as it was found significantly decreased in both *KRAS* mutant and *KRAS* wild-type CRC tissues (*p* = 0.0167 and *p* = 0.0367, respectively) (Fig. 4B), although it was more significant in *KRAS* mutant tissues.

### *BIRC5*/*Survivin* and *cIAP2* deregulation in CRC shows significant dependence on *KRAS* mutation status

*BIRC5*/*Survivin* gene was significantly up-regulated in *KRAS* wild-type CRC tissues (*p* = 0.042), but not in *KRAS*-mutant ones (*p* = 0.279) compared to normal pairs, as shown in Fig. 4C. Therefore, the over-expression of *BIRC5*/*Survivin* observed in CRC is particularly attributed to *KRAS* wild-type tumors.

*cIAP2* mRNA expression was significantly decreased in *KRAS-*mutant CRC tissues (*p* = 0.0001), but not in *KRAS* wild-type ones (*p* = 0.055) compared to normal pairs (Fig. 4D).

On the contrary, the use of Mann Whitney *U* test did not reveal statistically significant overexpression of *XIAP* or *DR4* in either group of cancerous tissues classified according to *KRAS* mutation status compared to normal pairs (Fig. 4E,F, respectively).

### Protein expression of apoptotic factors confirms the pattern of their mRNA expression in CRC: Protein expression of DR4, DR5, BIRC5/Survivin, is significantly upregulated in CRC. cIAP1 and cIAP2 protein levels are low in human colon cancer tissues

**Figure 5:**
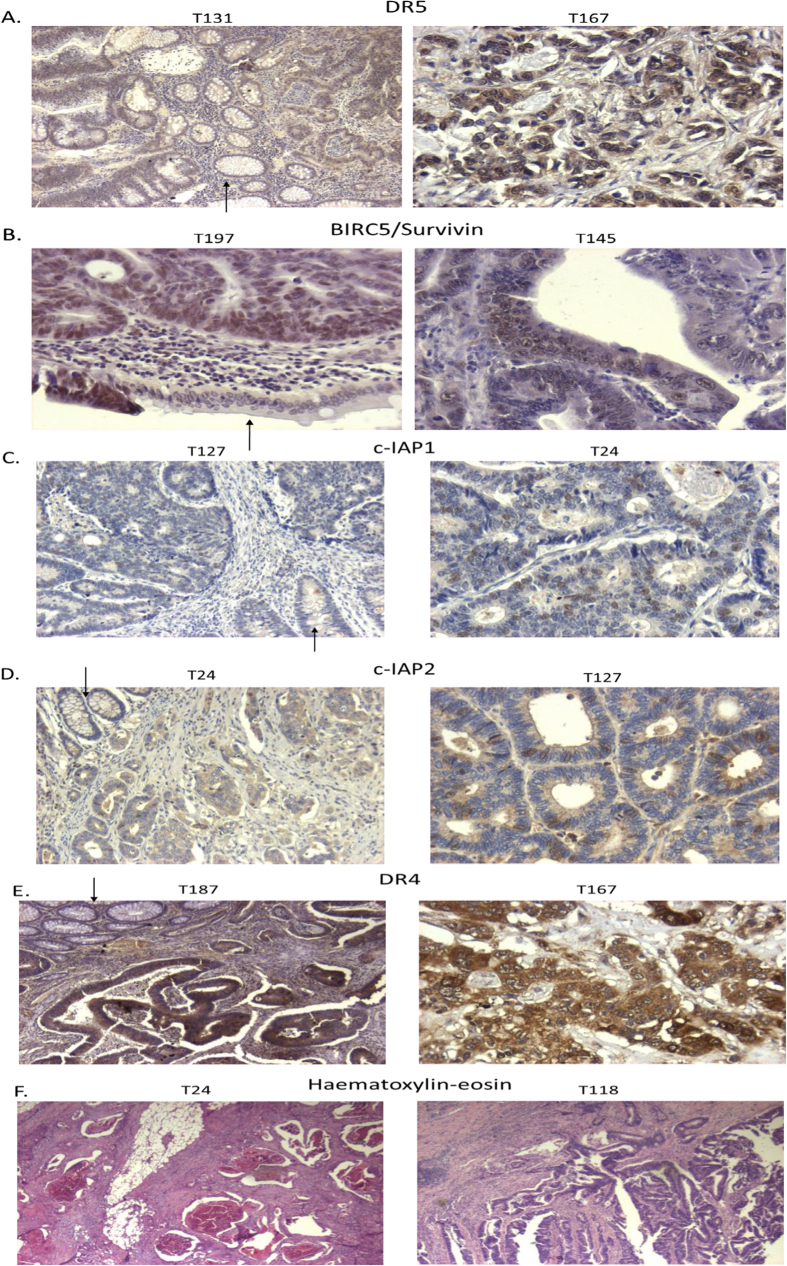
Analysis of protein expression and histological staining. Protein analysis for DR5 (**A**), BIRC5/Survivin (**B**), cIAP1 (**C**), cIAP2 (**D**) and DR4 (**E**) by immunohistochemistry in human colon tumors. Strong immunoreactivity is shown for DR5, DR4 in T167 and BIRC5/Survivin in T197. Moderate immunoreactivity is shown for BIRC5/Survivin in T145 and DR4 in T187. Mild immunoreactivity is shown for cIAP1 and cIAP2. Original magnification was 200X, except for T131, T127 for cIAP1, T24 for cIAP2 and T187, that was 100X. Arrows indicate normal mucosa. T: tumor. Haematoxylin-eosin (**H**,**E**) stained slides from the taken samples were used to confirm the percentage of tumor (**F**). Original magnification was 25X.

In the present study, protein expression of DR4, DR5, BIRC5/Survivin, cIAP1 and cIAP2 was determined immunohistochemically in twenty five colorectal cancer tissues that showed high levels of mRNA differential expression. All samples were found to express DR4, DR5 and BIRC5/Survivin showing either mild (+), moderate (++) or strong (+++) reactivity, whereas for cIAP1 and cIAP2, staining was either negative (−) or mild (+) (Table 3, Fig. 5). 23/25 (92%) colorectal cancer tissues were found to overexpress DR4, 19/25 (76%) were found to overexpress DR5 and 18/25 (72%) were found to overexpress BIRC5/Survivin (Table 3). For statistical analysis, the specimens that showed negative (−) or mild (+) staining were considered as 0 and those that showed moderate (++) or strong (+++) as 1. Normal mucosa for each respective tumor tissue was also considered as 0, since it showed negative reactivity. Paired samples t-tests were performed to evaluate the statistical significance of these results and in each case overexpression of the protein was found to be statistically significant (*p* < 0.0001). cIAP1 and cIAP2 protein expression was low (Table 3). In addition to protein expression analysis, sections from the tumor specimens were stained with haematoxylin-eosin to confirm the percentage of tumor. In all cases, the confirmed percentage of tumor ranged between 80 and 100 percent of the specimen, thus excluding cross-contamination from normal mucosa (Fig. 5F).

## Discussion

The use of molecular markers in cancer medicine is still evolving and it is clear that there is much yet to be clarified. In the last decades there is increasing interest in the use of molecular staging in CRC prognosis and treatment. The choice of the molecular markers remains the most prominent parameter. Given the fact that there is no such thing as the perfect marker, the best choice would include a combination of sensitive and specific markers, in order to achieve maximum clinical relevance.

### *DR5* gene is significantly up-regulated in CRC, particularly in TNM stage I/II tumors, including *KRAS*-mutant tumors

The TNF-Related Apoptosis Inducing Ligand, TRAIL, and its receptors in cancer have the role of a potent inducer of apoptosis in tumor cells while most normal cells are resistant. In the present study, expression of *DR4* and *DR5* was assessed in tumors from colorectal cancer patients in correlation to each patient’s normal mucosa in the absence of adjuvant chemotherapy, in association with colon cancer staging. *DR5* mRNA expression was found significantly higher in colorectal tumor specimens, as compared to physiological mucosa, especially in low TNM stage (I/II) CRC.

The identification of DR5 receptor up-regulation from early colon cancer stages may ensure better diagnosis for patients diagnosed early. Furthermore, DR5 expression suggests that, in addition to a potentially important role as a diagnostic but not prognostic marker, it might also have an important implication in therapy by administration of rhTRAIL and agonistic antibodies against DR5, already available.

Since *KRAS* mutation status in CRCs is correlated with resistance to treatment with anti-EGFR monoclonal antibodies, the effort for novel therapeutic approaches, such as TRAIL-related therapeutics, for *KRAS* mutated CRCs is crucial. Here, *DR5* mRNA expression was found significantly elevated in both *KRAS* mutant and *KRAS* wild-type CRC tissues as compared to their normal pairs, with greater significance in *KRAS* mutant specimens. Notably, oncogenic KRAS can regulate Death Receptors during metastasis in colorectal cancer cells^30^. The findings of this study, clearly propose that appropriate combinatorial therapeutic schemes involving recombinant TRAIL and/or agonistic antibodies against DR5 may be efficient against colorectal metastatic tumors, including those bearing *KRAS* mutations.

### *BIRC5*/Survivin and *cIAP2* genes are significantly deregulated in CRC, depending on the presence of *KRAS* mutations

*BIRC5*/Survivin over-expression has been reported to be involved in colorectal tumorigenesis by stimulating the transition of low-grade dysplasia adenomas into high dysplasia and CRC *in situ* and has been proposed as a biomarker with diagnostic and prognostic potential as well as a therapeutic target^6^,^27^,^31^. *BIRC5*/Survivin has been shown to be up-regulated by oncogenic c-H-Ras^32^. Moreover, the ratio between survivin and TRAIL receptors can be predictive of recurrent disease in neuroblastoma^33^. In this study, significant over-expression of *BIRC5*/Survivin in CRC is reported, which is particularly observed in *KRAS* wild-type tumors. The individual mechanism of differential regulation of *BIRC5*/Survivin by either mutant or wild-type *KRAS* needs to be further examined. Even when the mRNA levels not always translate to protein expression, there is good chance that tumors with up-regulated *BIRC5*/Survivin will be responsive to therapies based on specific inhibitors targeting their promoter and mRNA expression. Notably, a randomized open-label phase II study evaluating antitumor activity of the survivin antisense oligonucleotide LY2181308 (LY) in combination with docetaxel (DO) for second-line treatment of patients with non-small cell lung cancer (NSCLC) was established^34^. Moreover, a phase I/II study of sepantronium bromide (YM155, survivin suppressor) with paclitaxel and carboplatin in patients with advanced non-small-cell lung cancer has been reported^35^.

### *cIAP1* and *cIAP2* are significantly down-regulated in CRC and only *cIAP2* is dependent on *KRAS* mutation status; *cIAP2* down-regulation is significantly associated with lower survival

IAP family members have been shown to play an important role in apoptosis. Different IAP family members may be differentially involved in tumor progression, diagnosis and therapy, indicating the purpose of studies which examine the importance of individual IAP members. Moreover, individual IAP family members have been shown to be differentially regulated by *RAS* oncogene either directly or by a *RAS-*induced autocrine manner^36^. Here, *cIAP1* and *cIAP2* were found significantly down-regulated in CRC, and low expression of *cIAP2* was significantly correlated with detection of *KRAS* mutations in the colorectal tumors under study. Moreover, *cIAP2* down-regulation in CRC was found significantly associated with lower survival probability and shorter OS of CRC patients, thus demonstrating a potential prognostic usefulness. Relative expression of IAPs in a particular tumour can have a major impact on their diagnostic and/or prognostic capacity, as well as to therapies targeting apoptosis in cancer.

### Protein expression of apoptotic factors confirms the pattern of their mRNA expression in CRC

In an attempt to correlate mRNA expression levels with protein expression, immunohistochemistry was performed in a number of representative samples that were selected based on the levels of mRNA expression for each gene. Protein expression was confirmed in the investigated samples.

*DR5* was found to be significantly up-regulated in 76/100 CRC tissues and protein expression was also high in 19/25 (76%) samples analysed. *DR4* mRNA expression was elevated in CRC (59/100 patients), although not in a statistically significant manner, and protein expression was high in 23/25 (92%) investigated tumors, with a statistical significance. *BIRC5*/Survivin was significantly up-regulated in the 100 CRC tissues examined (61/100) and protein expression was also significantly high in 18/25 (72%) CRC tissues. *cIAP1* and *cIAP2* were found to be significantly down-regulated in CRC (67/100 and 69/100 respectively) and protein expression was low in all samples analysed.

### 5-genes biomarker panel (*DR5*, *BIRC5/*Survivin, *XIAP*, *cIAP1* and *cIAP2*) with significant differential diagnostic potential

Differential mRNA expression of 5 genes *DR5*, *XIAP*, *BIRC5*/Survivin, *cIAP1* and *cIAP2* between CRC and normal colorectal tissue is reported here. Data were combined using logistic regression and 5-gene panel ROC analysis was performed on all samples in order to evaluate overall discriminatory value of the 5 genes expression levels between CRC and normal tissues. There is very significant fair discriminatory value of the 5-gene panel between CRC and normal tissues which could have significant differential diagnostic value.

This study confirms the central role of apoptosis pathways in CRC, despite the fact that the molecular mechanism remains unclear. The mRNA expression profiling of 5 apoptosis genes could contribute to CRC diagnosis, in cooperation with known molecular, clinical, and histologic diagnostic factors. In case that this promising result can be valid in a much larger patient cohort, the apoptotic signature could not only enhance the diagnostic accuracy to identify early stage CRC, but could also provide good candidate cancer diagnostic/predictive biomarkers and/or putative drug targets for tumor therapies. Therefore, further evidence is necessary towards this direction.

## Methods

### Study group

In this study, 200 fresh frozen colorectal tissues obtained from 100 patients with sporadic CRC collected at the G. Gennimatas General Hospital of Athens were examined. CRC specimens, 0.5–2 cm in diameter, were acquired from patients who underwent surgery for primary infiltrating CRC. Tumor specimens were taken from the most central part of the mass, thus ruling out the possibility of cross-contamination from normal mucosa or adenomatous tissue. Sections from the tumor specimens were stained with haematoxylin-eosin to confirm the percentage of tumor. In all cases, the confirmed percentage of tumour ranged between 80 and 100 percent of the specimen, thus excluding cross-contamination from normal mucosa (Fig. 5F). At the same time, adjacent normal colonic mucosa, sufficiently separated from the tumor, was also collected. Normal colonic tissue was taken from the histologically cancer-free margins of the resected colon to ensure absence of cancerous cells. The remaining biopsy section was prepared for routine histology. All specimens were processed immediately after surgical resection. Patients with low-lying locally advanced rectal cancers who received neoadjuvant chemo-/radiotherapy were excluded from the study to avoid possible bias deriving from unknown radiation-mediated effects on tumor gene status. The mean age of patients was 69.5 ± 1.2 years (ranging from 42 to 95). Informed consent was obtained from all patients. The study protocol was approved by the Medical Ethical Review Committee of the G. Gennimatas General Hospital of Athens (Institutional Review Board number: 4/06). Clinical and histological information for the tumor samples included in the present study (Table 4) was obtained and used for statistical analyses. Preoperative clinical staging was accomplished by physical examination (hepatomegaly, ascites, palpable tumors, lymphadenopathy), CT scan of the abdomen and pelvis and chest imaging. Staging was performed according to the Tumor Node Metastases (TNM) staging system (7^th^ edition) of the American Joint Committee on Cancer/Union for International Cancer Control^37^. Out of the 100 CRC tissues included in the study, 50 were *KRAS* wild-type and 50 were *KRAS*-mutant tissues, harboring the activating *KRAS-G13D*, *KRAS-G12D* and *KRAS-G12V* mutations. Research was carried out in accordance with the ethical standards of the Helsinki Declaration of 1995, as revised in Tokyo 2004.

### RNA extraction and Reverse transcription

Upon collection, colon tissue samples were immersed in RNA*later*^®^ solution (Ambion by Life Technologies, Austin, TX, USA) and transferred to our laboratory at 4 °C. Cancerous and normal colon tissues were homogenized in 2 ml TRIzol reagent (Invitrogen by Life Technologies, Carlsbad, CA, USA) on ice using an electric tissue grinder (ULTRA-TURRAX, type T-25, Junke and Kunkel). Total RNA was extracted from homogenized tissue according to the manufacturer’s instructions. Five micrograms of total RNA were reverse transcribed into first-strand cDNA using oligo(dT) primer, with the SuperScript^TM^ II Reverse-Trascriptase pre-amplification system (Invitrogen by Life Technologies, Carlsbad, CA, USA), following the manufacturer’s instructions.

### Quantitative real time PCR

Singleplex fluorescent-based quantitative real-time PCR assay (qPCR) was performed for the determination of *DR4*, *DR5*, *cIAP1*, *cIAP2*, *XIAP* and *BIRC5* mRNA expression levels in the colon specimens on an iQ5 Real Time PCR Detection System (Bio-Rad Laboratories, Inc., USA). The quantitative PCR analysis was performed in 96-well plates using iQ SYBR Green Supermix (Bio-Rad Laboratories, Inc., USA). The 10 ml reaction mixtures contained 75 ng cDNA, 50 nM primers, and 5 μl iQ SYBR Green Supermix. The thermal protocol consists of a 10 min polymerase activation step at 95 °C, followed by 40 cycles of denaturation at 95 °C for 20 sec and the primer annealing and extension at 60 °C for 1 min. Each sample was amplified twice in duplicates and the average C_T_ values were calculated for the expression analysis. Following the amplification, a dissociation curve was generated in order to verify the PCR products of interest from any non-specific ones or any primer-dimer production, by their specific melting temperature (Tm). Relative quantification analysis for the *DR4*, *DR5*, *cIAP1*, *cIAP2*, *XIAP* and *BIRC5*/Survivin mRNA expression was performed with the comparative 2^−ΔΔCT^ method. mRNA expression of all genes was normalized with the use of the *GAPDH* and *HPRT* reference genes, while the Caco-2 cell line was used as a calibrator. The primers used for each gene were the following: *GAPDH* (NM_002046) F: 5′-GAAGGTGAAGGTCGGAGT-3′, R: 5′-CATGGGTGGAATCATATT-3′, amplicon 155 bp. *HPRT* (NM_000194.2) F: 5′-TGACACTGGCAAAACAATGCA-3′, R: 5′-GGTCCTTTTCACCAGCAAGCT-3′, amplicon 94 bp. *DR4* (NM_003844.3) F: 5′-TCCAGCAAATGGTGCTGAC-3′, R: 5′-GAGTCAAAGGGCACGAT-3′, amplicon 77 bp. *DR5* (NM_003842.4) F: 5′-CCAGCAAATGAAGGTGATCC-3′, R: 5′-GCACCAAGTCTGCAAAGTCA-3′, amplicon 66 bp. *XIAP* (NM_001167.3) F: 5′-GACAGTATGCAAGATGAGTCAAGTCA-3′, R: 5′-GCAAAGCTTCTCCTCTTGCAG-3′, amplicon 92 bp. *cIAP1*/*BIRC2* (NM_001166.4) F: 5′-TGTTGTCAACTTCAGATACCACTGG-3′, R: 5′-CATCATGACAGCATCTTCTGAAGA-3′, amplicon 97 bp. *cIAP2*/*BIRC3* (NM_001165.4) F: 5′-TCCGTCAAGTTCAAGCCAGTT-3′, R: 5′-TCTCCTGGGCTGTCTGATGTG-3′, amplicon 70 bp. *BIRC5*/Survivin (NM_ 001012271.1) F: 5′-GCACGGTGGCTTACGCCTG-3′, R: 5′-AACCGGACGAATGCTTTTTATGTTCC-3′, amplicon 90 bp.

### Immunohistochemistry

Protein expression of DR4, DR5, BIRC5/Survivin, cIAP1 and cIAP2 in human colon tumors was determined immunohistochemically in twenty five representative specimens, in order to confirm the respective findings from the mRNA expression analysis. Samples for this part of the analysis were selected based on the levels of mRNA expression for each gene under study. For histology, 4-μm sections were cut from formalin-fixed, paraffin-embedded tissues and stained with haematoxylin-eosin. For the detection of DR4, a rabbit polyclonal antibody (ProSci Inc., Poway, CA, USA) was applied on serial paraffin sections at 5 μg/ml for 1hr at room temperature, whereas for the detection of DR5 a rabbit polyclonal antibody (ProSci Inc., Poway, CA, USA) was applied at 1 μg/ml for 1 hr at room temperature. BIRC5/Survivin was detected using a rabbit monoclonal antibody (Cell Signaling Technology, Danvers, MA, USA) on serial paraffin sections at 1 μg/ml for 1hr at room temperature. For the detection of cIAP1 and cIAP2, a rabbit polyclonal antibody (Santa Cruz Biotechnology, Inc., Dallas, TX, USA) was applied separately on serial paraffin sections at 4 μg/ml for 1 hr at room temperature, after antigen unmasking using PT Link (pH 6) for Pre-Treatment (DAKO, Glostrup, Denmark) for 15 mins. The Envision polymer detection kit (DAKO, Glostrup, Denmark) was utilized and the 3′,3′-diaminobenzidine was used as chromogen to reveal the antigen binding sites in all cases. Staining intensity was divided in three classes (+, ++, +++). Sections from the same specimen, where the primary antiserum was substituted by buffer solution, served as negative controls. Positive controls were used following manufacturer’s instructions. Normal mucosa was also stained for each corresponding tumor specimen. WHO histological criteria were used for the histological classification of adenocarcinomas.

### Statistical analysis

Differences in the mRNA expression profile of the six genes between CRC and normal tissue specimens were evaluated with the non-parametric Wilcoxon Signed Ranks test. CRC tissues were classified according to their clinico-pathological features (TNM stage, lymph node invasion status) and statistical analysis was performed with the use of Mann-Whitney *U* test. Receiver-operating characteristic (ROC) curves were constructed for quantitative PCR data sets using a single-gene test in CRC versus normal tissues, by plotting sensitivity versus (1-specificity). Quantitative data demonstrating differential mRNA expression between CRC and normal tissue were combined using logistic regression and the performance of a multi-gene panel ROC analysis was performed on all samples. The areas under the ROC curves (AUC) were analysed by Hanley and McNeil method. Moreover, differential mRNA expression profile of the six genes between CRC and normal tissues was examined in subgroups of patients according to *KRAS* mutation status (mutant or wild-type CRC tissue) using the Mann-Whitney *U* test. A p-value of 0.05 (or less) was considered as statistically significant. Statistical analyses were performed using the MedCalc for Windows software, version 15.6 (MedCalc Software, Ostend, Belgium). Survival analysis was performed by constructing Kaplan-Meier overall survival (OS) curves for high- and low- gene expression of patients for the six genes under study and the Wilcoxon Signed log-rank test was used to compare survival between subgroups of patients. Cutoff finder^38^ was implemented to generate an optimal cut-off point for each gene under study, as there are no established cut-off points concerning their expression in CRC. A p-value of 0.05 (or less) was considered as statistically significant.

## Additional Information

**How to cite this article**: Devetzi, M. *et al.* Death receptor 5 (*DR5*) and a 5-gene apoptotic biomarker panel with significant differential diagnostic potential in colorectal cancer. *Sci. Rep.* **6**, 36532; doi: 10.1038/srep36532 (2016).

**Publisher’s note**: Springer Nature remains neutral with regard to jurisdictional claims in published maps and institutional affiliations.

## Supplementary Material

Supplementary Information

## Figures and Tables

**Table 1:** *DR* and *IAP* genes mRNA expression in paired colorectal tissues.

Variable	Mean ± SEM^a^	Range	Percentiles			
Fold mRNA expression			10	25	50 (median)	75
***DR5***						
Cancerous (N = 100)	1.68 ± 0.11	0.243–7.608	0.645	0.979	1.395	2.149
Normal (N = 100)	0.979 ± 0.05	0.172–2.66	0.435	0.648	0.846	1.172
***DR4***						
Cancerous (N = 100)	1.926 ± 0.13	0.135–7.23	0.806	1.108	1.641	2.267
Normal (N = 100)	1.661 ± 0.08	0.245–4.62	0.85	1.128	1.429	2.176
**Survivin/*****BIRC5***						
Cancerous (N = 100)	0.606 ± 0.06	0.043–4.55	0.142	0.238	0.383	0.762
Normal (N = 100)	0.421 ± 0.04	0.061–2.64	0.146	0.205	0.304	0.425
***XIAP***						
Cancerous (N = 100)	1.165 ± 0.03	0.182–5.026	0.344	0.615	0.999	1.533
Normal (N = 100)	0.986 ± 0.06	0.129–3.658	0.406	0.621	0.884	1.211
***cIAP1***						
Cancerous (N = 100)	1.212 ± 0.07	0.103–5.186	0.431	0.745	1.045	1.488
Normal (N = 100)	1.610 ± 0.09	0.271–6.788	0.806	1.016	1.350	1.880
***cIAP2***						
Cancerous (N = 100)	5.652 ± 0.53	0.305–29.119	1.063	2.018	3.557	7.165
Normal (N = 100)	9.208 ± 0.93	1.421–55.123	2.929	4.077	6.280	10.093

^a^SEM: Standard Error of the Mean.

**Table 2:** *DR* and *IAP* genes mRNA expression analysis in pairs of primary cancerous and normal colorectal tissues.

Variable	Number of patients	*p* value*
***DR5***		
Higher in cancer vs normal	81	
Lower in cancer vs normal	18	**<0.0001**
Approx. equal in both tissues	1	
***DR4***		
Higher in cancer vs normal	59	
Lower in cancer vs normal	35	0.5485
Approx. equal in both tissues	6	
***BIRC5*****/Survivin**		
Higher in cancer vs normal	64	
Lower in cancer vs normal	33	**0.0002**
Approx. equal in both tissues	3	
***XIAP***		
Higher in cancer vs normal	58	
Lower in cancer vs normal	39	**0.0300**
Approx. equal in both tissues	3	
***cIAP1***		
Higher in cancer vs normal	25	
Lower in cancer vs normal	69	**<0.0001**
Approx. equal in both tissues	6	
***cIAP2***		
Higher in cancer vs normal	30	
Lower in cancer vs normal	69	**<0.0001**
Approx. equal in both tissues	1	

^*^Calculated by the non-parametric Wilcoxon Signed Ranks Test.

**Table 3:** Protein expression of DR4, DR5, BIRC5/Survivin, cIAP1 and cIAP2 by immunohistochemistry.

Tumor samples	PROTEIN EXPRESSION OF DR4, DR5, cIAP1, cIAP2 and BIRC5/Survivin BY IMMUNOHISTOCHEMISTRY							
	DR4	DR5	cIAP1	cIAP2	BIRC5/Survivin	*KRAS* mutation status	Differentiation	TNM
T24	+++	+++	+	+	++	*KRAS* mutant	Middle-low	T3N2M1
T108	+++	++	+	+	++	*KRAS* mutant	N/A	T3N0M0
T113	++	++	+	+	+++	*KRAS* wild type	Middle	T2N1M0
T114	+	+	−	−	+	*KRAS* mutant	N/A	N/A
T118	++	+	−	+	+	*KRAS* wild type	Middle	T3N2M0
T121	++	+	−	−	+	*KRAS* mutant	N/A	N/A
T127	++	+	+	+	++	*KRAS* mutant	Middle	T1N1M0
T131	++/+++	++/+++	+	+	++	*KRAS* wild type	Middle	T2N1M0
T132	+++	++	+	+	++	*KRAS* mutant	Middle	T2N1M1
T139	+++	+++	+	+	++	*KRAS* wild type	Middle	T3N0M0
T140	+	+/++	−	−	+/−	*KRAS* mutant	N/A	N/A
T145	+++	++	+	+	++	*KRAS* mutant	Middle	T3N1M0
T151	+++	+++	+	+	+++	*KRAS* mutant	Middle	T3N1M0
T166	+++	++	+/−	−	++	*KRAS* wild type	Low	T2N0M0
T167	+++	+++	+	+	++	*KRAS* wild type	Middle	T3N1M1
T178	+++	+++	+	+	++	*KRAS* wild type	Middle	T3N1M0
T182	+++	++	+	+	++	*KRAS* wild type	Low	T3N0M0
T185	++	++	−	+	++	*KRAS* mutant	Middle	T3N2M1
T189	+++	+++	+/−	+	+++	*KRAS* mutant	Low	T3N1M0
T193	+++	+++	+	+	++	*KRAS* mutant	Middle	T3N2M0
T194	++	+	+/−	+	+	*KRAS* mutant	Middle	T3N0M0
T196	+++	+++	+	+	+++	*KRAS* mutant	Middle	T3N1M0
T197	+++	++	−	+	+++	*KRAS* mutant	Low	T3N0M0
T200	++	++	−	+	+	*KRAS* mutant	Middle	T3N0M0
T201	++	+	+/−	+	+	*KRAS* mutant	Middle	T3N2M1

T: tumor; +: mild reactivity; ++: moderate reactivity; +++: strong reactivity; −: negative reactivity; N/A: information not available.

**Table 4:** Patient cohort characteristics.

**Age**	
Range (Mean ± SEM)	42–95 (69,5 ± 1,2)
**Gender**	
Male	59
Female	41
**Location**	
Descending colon	7
Right colon (cecum, ascending colon)	14
Transverse	5
Sigmoid	13
Rectum/Rectosigmoid	61
**Differentiation**	
High	9
Moderate	72
Low/Middle-low	14
Not available	5
**TNM Stage**	
I	22
II	30
III	30
IV	18

SEM: Standard Error of the Mean.
