# Supplementary material for: Death receptor 5 (DR5) and a 5-gene apoptotic biomarker panel with significant differential diagnostic potential in colorectal cancer
Source: Sci Rep. 2016 Nov 9;6:36532. doi: 10.1038/srep36532 (PMC5101514; doi:10.1038/srep36532)

Death receptor 5 *(DR5)* and a 5-gene apoptotic biomarker panel with significant differential diagnostic potential in colorectal cancer.

Marina Devetzi[1], Vivian Kosmidou[1], Margarita Vlassi[1], Iraklis Perysinakis[2], Chrysanthi Aggeli[2], Theodosia Choreftaki3, Georgios N. Zografos[2], Alexander Pintzas[1]*

Supplementary Figure 1. Box plots show the mRNA expression of DR4 (A), BIRC5/Survivin (C), XIAP (E) and cIAP2 (G) in TNM stage-classified CRC tissues and of DR4 (B), BIRC5/Survivin (D), XIAP (F) and cIAP2 (H) in lymph node invasion status-classified CRC tissues. Quartiles (25th, 50th-median, 75th percentiles) are within the box. The horizontal lines indicate the medians. The upper and nether horizontal lines indicate the 90th and 10th percentiles, respectively. Differences in mRNA expression were not significantly different between groups of patients classified according to TNM stage or lymph node invasion status.

Supplementary Figure 2. Representative melting curves for GAPDH (A), HPRT (B), DR5 (C), DR4 (D), BIRC5/Survivin (E), XIAP (F), cIAP1 (G) and cIAP2 (H).

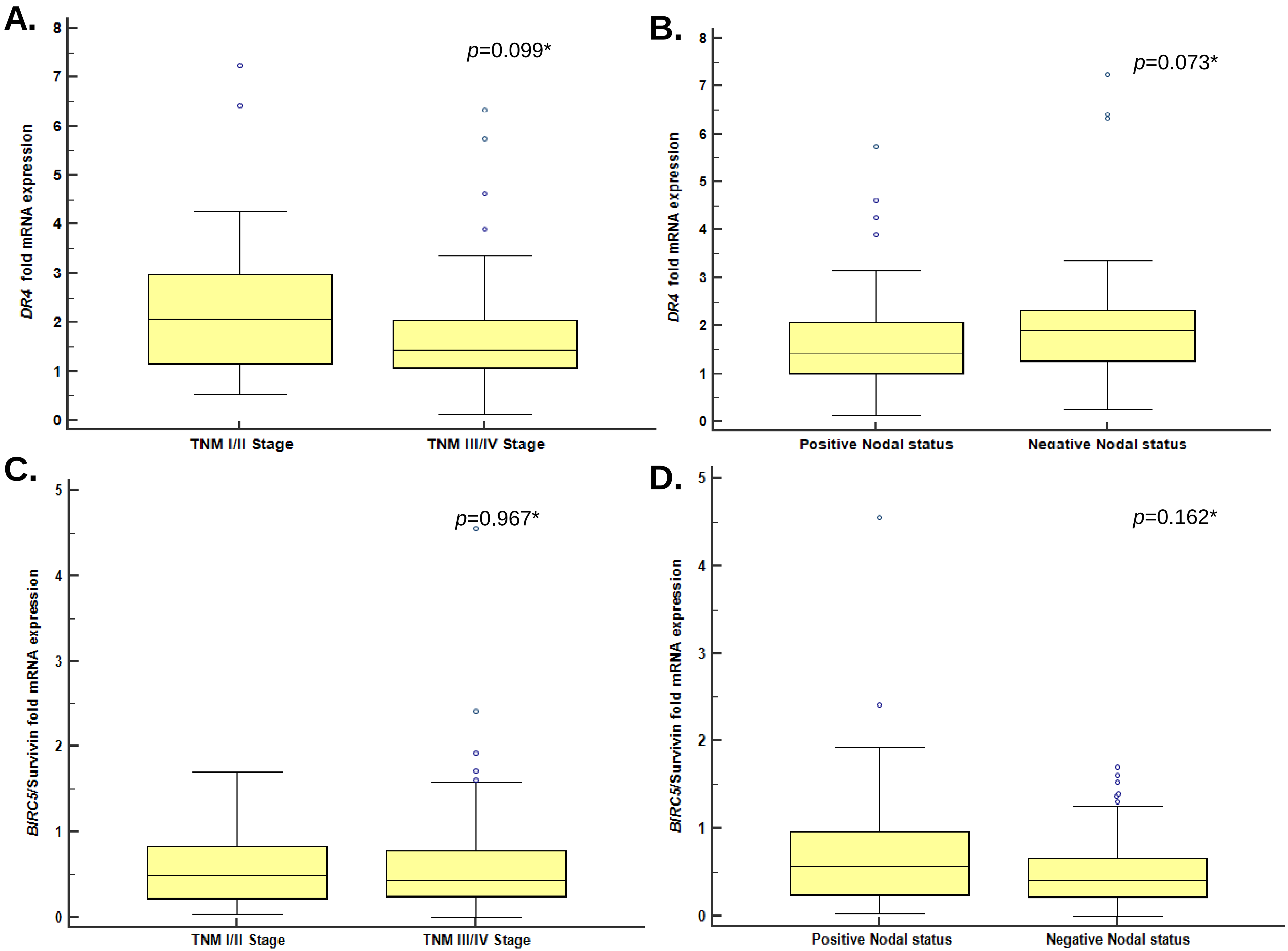

A.
DR4 fold mRNA expression
p=0.099*
TNM I/II Stage
TNM III/IV Stage
B.
DR4 fold mRNA expression
p=0.073*
Positive Nodal status
Negative Nodal status
C.
BIRC5/Survivin fold mRNA expression
p=0.967*
TNM I/II Stage
TNM III/IV Stage
D.
BIRC5/Survivin fold mRNA expression
p=0.162*
Positive Nodal status
Negative Nodal status

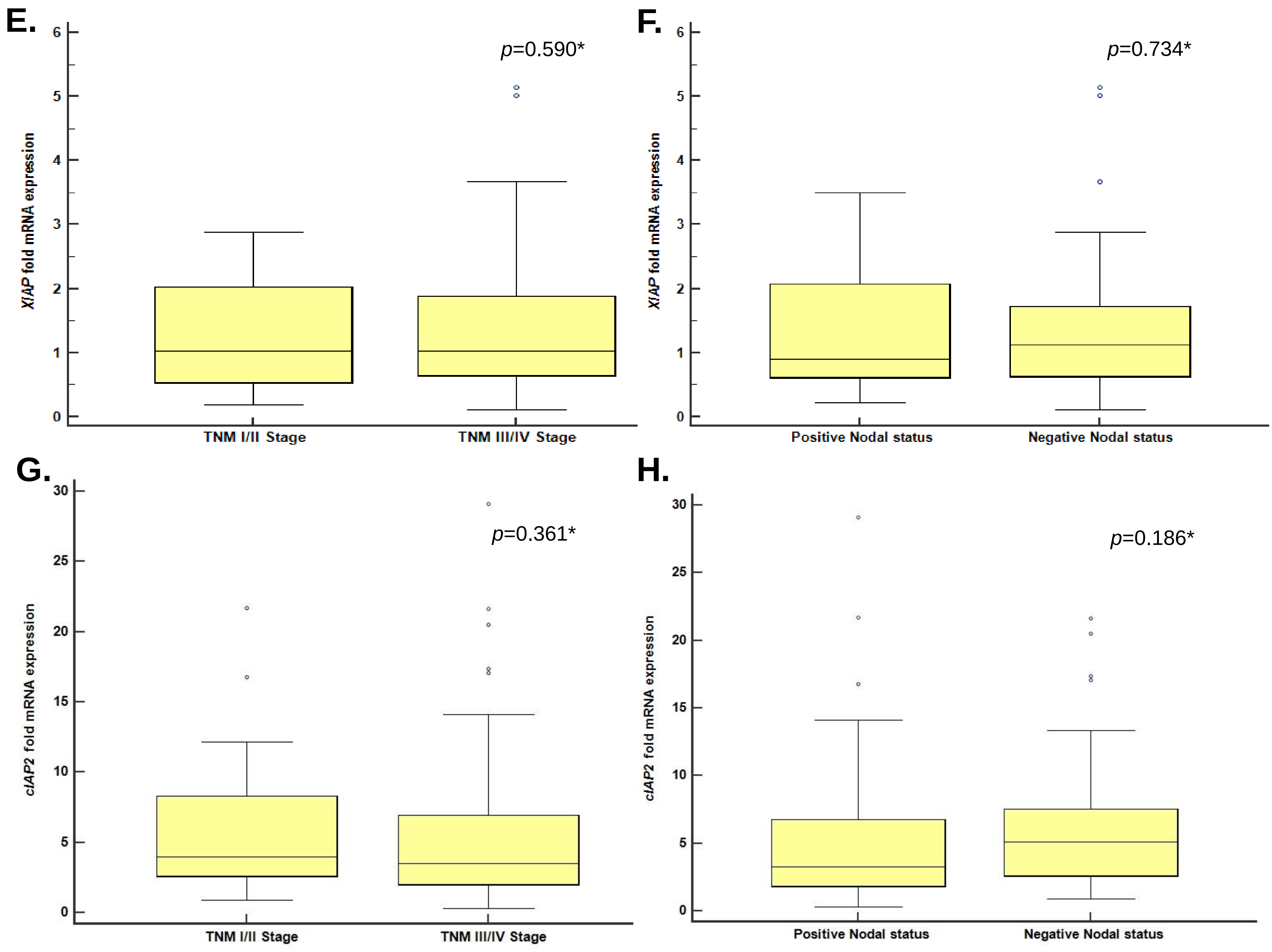

*Calculated by the Mann Whitney *U* test; The bold line bars represent the median value (50th percentile).

Representative melting curves for each gene

A.

GAPDH

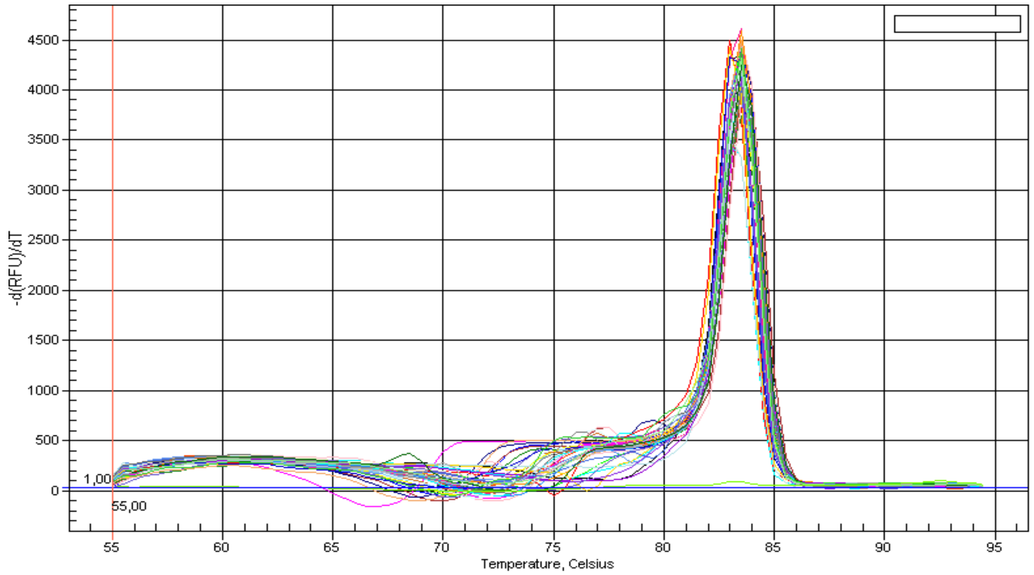

B.

HPRT

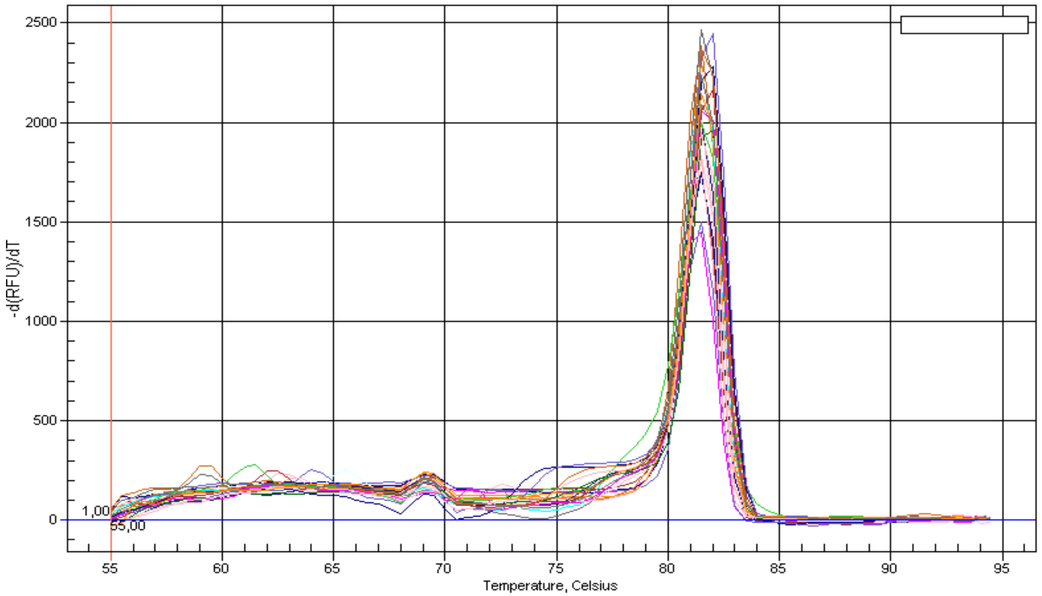

C.

DR5

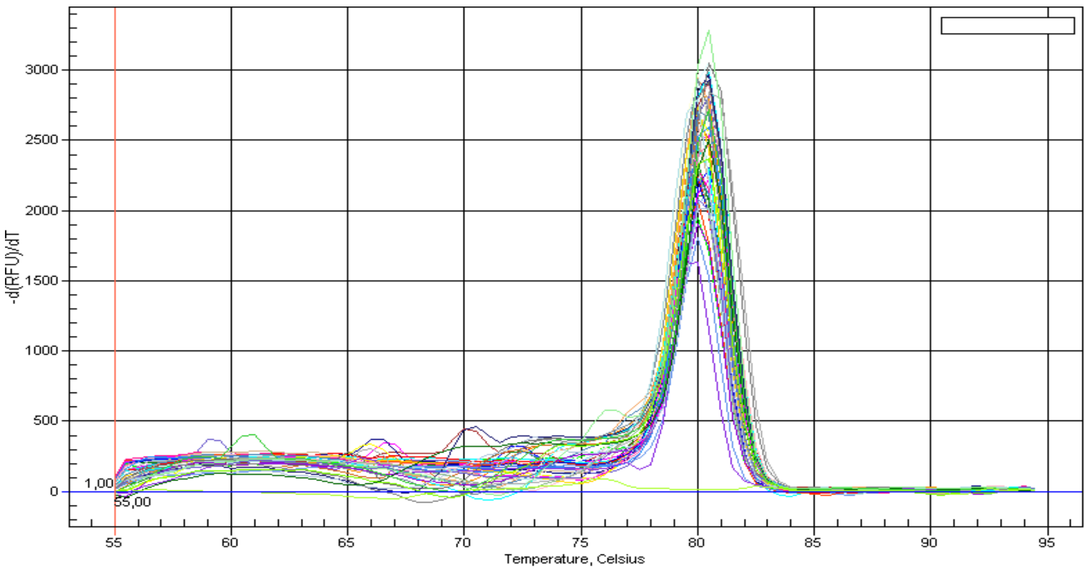

-d(RFU)/dT
3000
2500
2000
1500
1000
500
0
1,00
55,00
55
60
65
70
75
80
85
90
95
Temperature, Celsius

D.

DR4

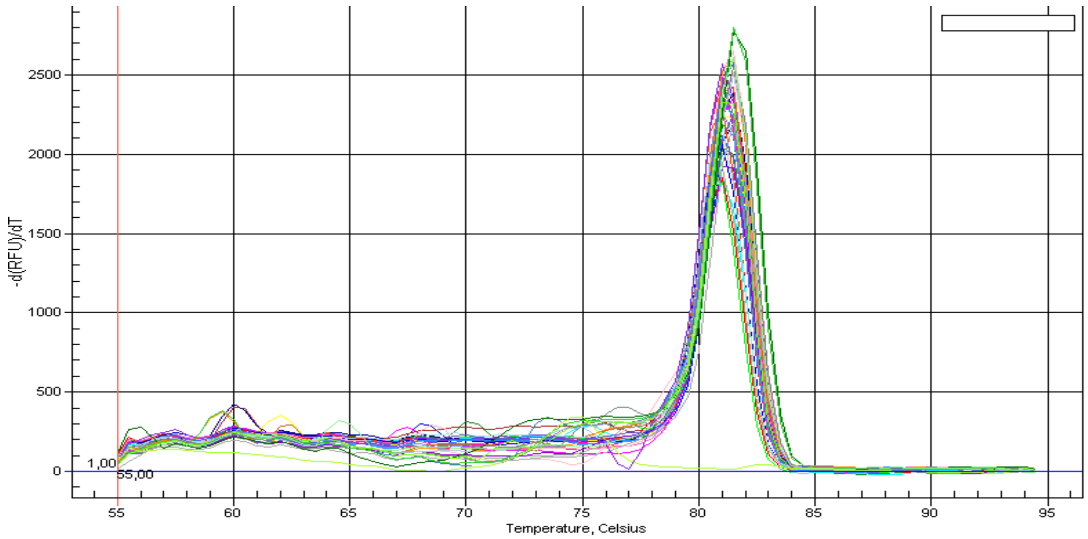

-d(RFU)/dT
2500
2000
1500
1000
500
0
1,00
55,00
55
60
65
70
75
80
85
90
95
Temperature, Celsius

E.

BIRC5/Survivin

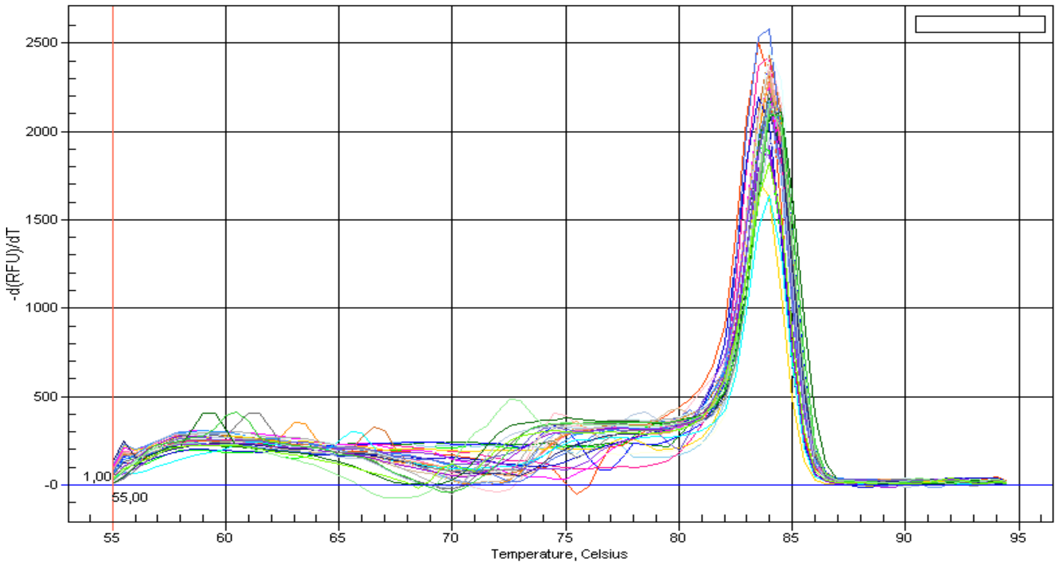

F.

XIAP

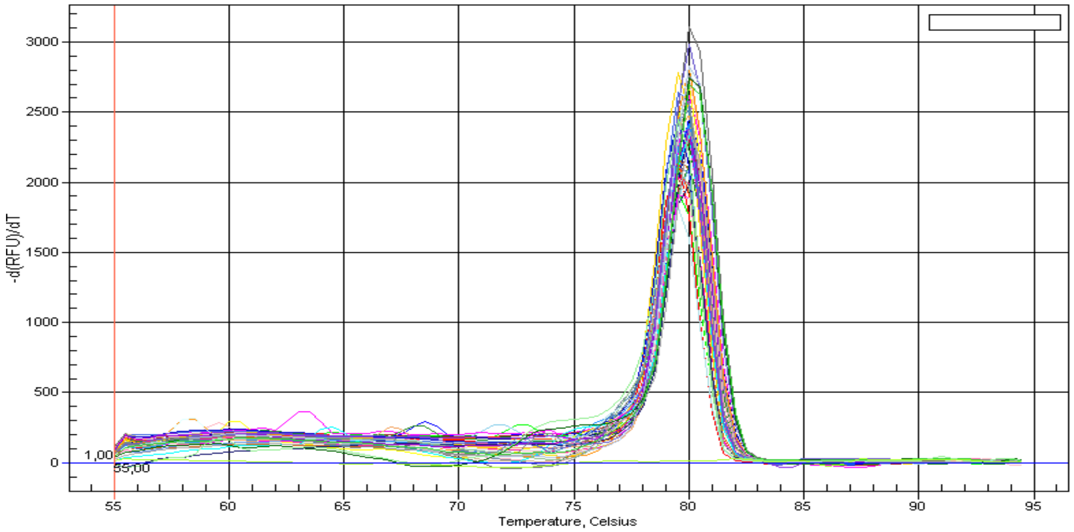

G.

cIAP1

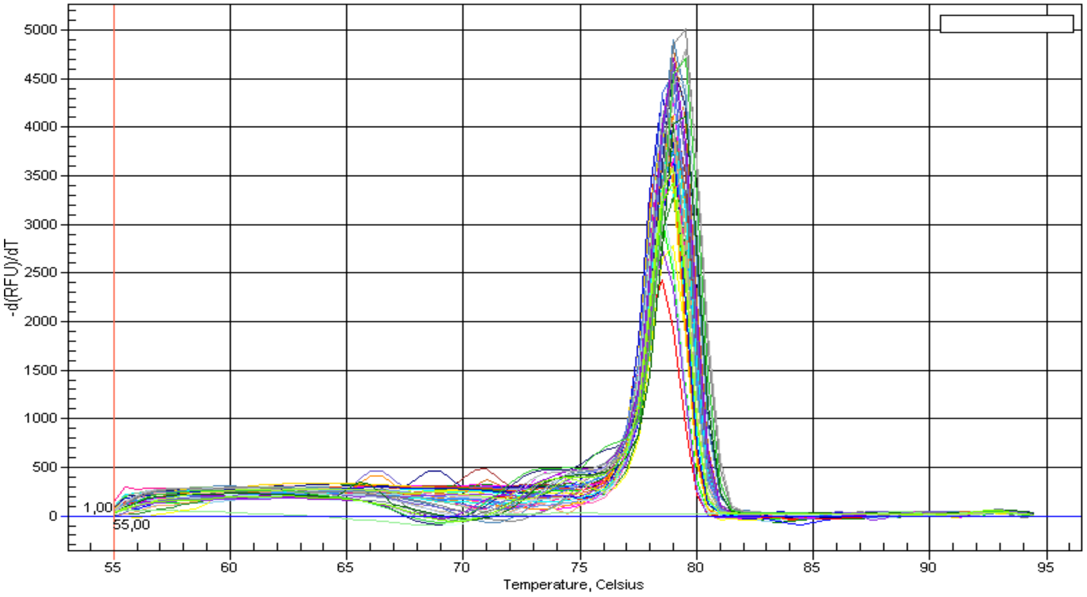

H.

cIAP2

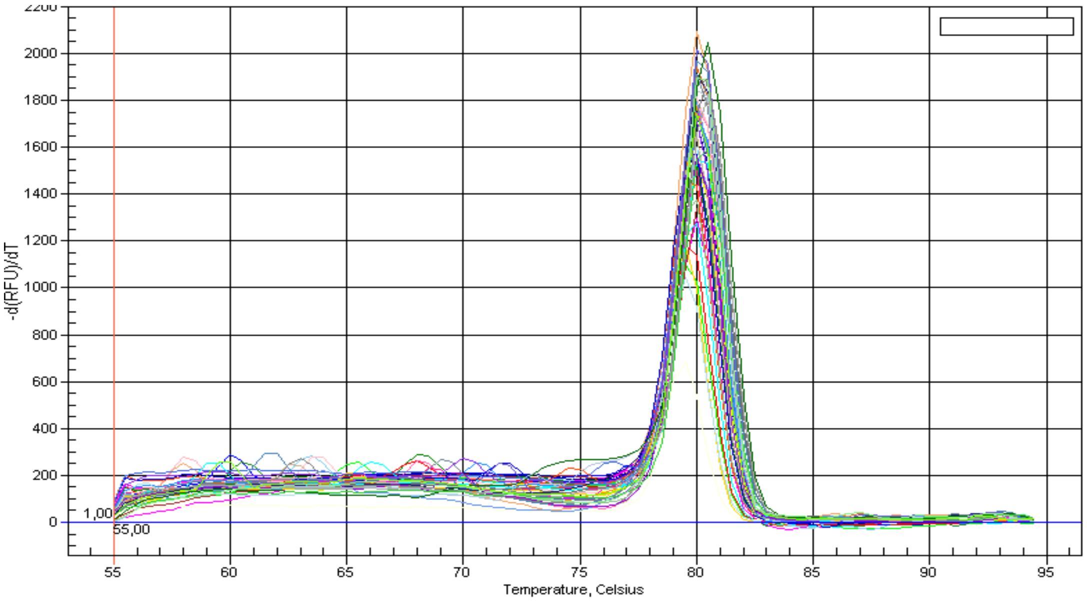

Supplement: Supplementary Information [file srep36532-s1.pdf]
